# Fermentation Products of Solvent Tolerant Marine Bacterium *Moraxella* spp. MB1 and Its Biotechnological Applications in Salicylic Acid Bioconversion

**DOI:** 10.1371/journal.pone.0083647

**Published:** 2013-12-31

**Authors:** Solimabi Wahidullah, Deepak N. Naik, Prabha Devi

**Affiliations:** Bioorganic Chemistry Lab, Chemical Oceanography Division, CSIR- National Institute of Oceanography, Dona Paula, Goa, India; University of Iowa, United States of America

## Abstract

As part of a proactive approach to environmental protection, emerging issues with potential impact on the environment is the subject of ongoing investigation. One emerging area of environmental research concerns pharmaceuticals like salicylic acid, which is the main metabolite of various analgesics including aspirin. It is a common component of sewage effluent and also an intermediate in the degradation pathway of various aromatic compounds which are introduced in the marine environment as pollutants. In this study, biotransformation products of salicylic acid by seaweed, *Bryopsis plumosa,* associated marine bacterium, *Moraxella* spp. MB1, have been investigated. Phenol, conjugates of phenol and hydroxy cinnamic acid derivatives (coumaroyl, caffeoyl, feruloyl and trihydroxy cinnamyl) with salicylic acid (3–8) were identified as the bioconversion products by electrospray ionization mass spectrometry. These results show that the microorganism do not degrade phenolic acid but catalyses oxygen dependent transformations without ring cleavage. The degradation of salicylic acid is known to proceed either via gentisic acid pathway or catechol pathway but this is the first report of biotransformation of salicylic acid into cinnamates, without ring cleavage. Besides cinnamic acid derivatives (9–12), metabolites produced by the bacterium include antimicrobial indole (13) and β-carbolines, norharman (14), harman (15) and methyl derivative (16), which are beneficial to the host and the environment.

## Introduction

Salicylic acid (SA) is a key intermediate in the catabolism of PAHs, naphthalene, naphtaquinone, phenanthrene and fluorene [1], [2]. It is widely produced by plants and some bacterial genera such as *Pseudomonas* and *Vibrio* spp. [3]. In several bacteria salicylate is active as siderophore [3] and also plays a role in gene regulation such as the expression of antibiotic resistance [4]. SA and its derivatives, particularly acetylsalicylic acid, are commonly used as effective analgesics and are available to the public in a wide variety of formulations [5]. Pharmaceuticals are released into the environment through human excretion, agricultural run-off, and wastewater from sewage treatment plants and pharmaceutical manufacturers [6].

The degradation of SA is known to proceed either via gentisic acid pathway or catechol pathway. *Pseudomonas* spp. is known to degrade salicylate by oxidative decarboxylation to produce catechol, the key intermediate being salicylate-1-hydroxylase [7], [8]. The hydroxylation of salicylic acid at the C-5 position by salicylate-5-hydroxylase to yield gentisic acid has also been observed in *Rhodococcus* [9], *Lignobacter* [10] and *Micrococcus* spp. [11]. Grund et al. [12] demonstrated the existence of two different routes within the genera *Streptomyces*. *S. olivaceiscleroticus* DSM415595, and *S. niger* DSM40302 converted SA to catechol. However, *S. umbvinus* DSM40278 converted SA to gentisic acid. Salicylate-1-hydroxylase is one of the model enzymes for flavin containing monooxygenase [13] while salicylate-5-hydroxylase which is responsible for the formation of gentisic acid intermediate which requires unusual co-factors CoA and ATP [14]. There is yet another group who opine that *Moraxella* spp. strain VG45 isolated from oil field samples degraded SA via salicylate-5-hydroxylase, gentisate 1,2 dioxygenase and then by a glutathione independent maleyl pyruvate hydrolase [15]. In the current study SA is not degraded by *Moraxella* spp. strain MB1 instead SA undergoes esterification with phenolic acids, metabolites produced by the strain MB1.

The main objective of the present investigation is to study the role of the marine bacterium *Moraxella* MB1 in the bioconversion of salicylic acid in the marine environment, where it is released as pollutant. In our earlier communication [16], we have reported decarboxylation of a nephrotoxin, citrinin, into decarboxycitrinin by the seaweed (*Bryopsis plumosa*) associated marine bacterium *Moraxella* spp. MB1. In the present study, metabolism of SA by the same bacterium has been studied and the extracellular metabolites identified using electrospray ionization (ESI-MS) and tandem mass spectrometry (ESI-MS/MS). Phenol and conjugates of phenol and hydroxy cinnamic acid derivatives (coumaroyl, caffeoyl, feruloyl and trihydroxy cinnamyl) with SA (3–8) were identified as the products of bioconversion. In order to obtain a better understanding of the metabolism of SA, metabolites produced by the bacterium have also been analysed. Besides cinnamic acid derivatives (9–12) the bacterium was found to produce adenine, guanine, and antimicrobial indole (13) and β-carbolines (14–16). Based on the present findings, probable metabolic pathways have been postulated for SA bioconversion products by *Moraxella* spp. strain MB1.

## Results

### Identification of Fermentation Products of *Moraxella* spp. MB1 with SA (in Biphasic Medium)

#### NMR analysis

**Figure 1:**
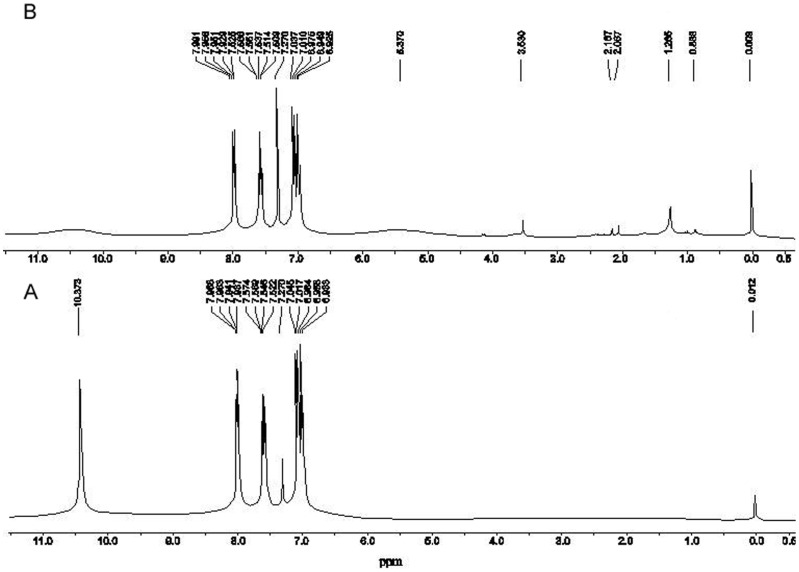
^1^H NMR spectra of control salicylic acid (A) and its bioconversion products (B).

Examination of the proton NMR spectra of the products in the control flask (without culture, Figure 1A) and the bio- transformed products from the experimental flask (inoculated with *Moraxella* spp. MB1, Figure 1B) indicated that the sharp singlet due to carboxyl proton of salicylic acid at δ 10.37 (in control), is replaced by a broad hump in the spectrum of experimental product. The broad signal probably results from the carboxyl protons in different environment in different molecules and as the concentration of these molecules is low, the signal is broad. An additional broad signal at δ 5.37 evident in the spectrum of transformed product (Figure 1B) was attributed to the olefenic protons of the acrylic moiety present in CA derivatives.

#### ESI-MS analysis

**Figure 2:**
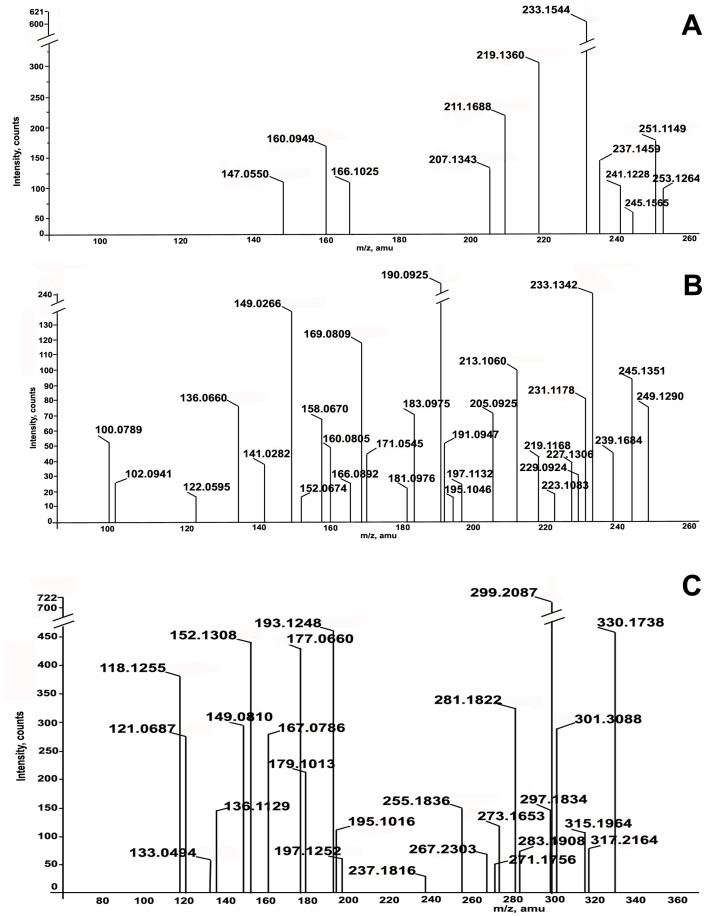
Negative ESI-MS profile of products of fermentation of *Moraxella* spp. MB1 in aqueous medium without salicylic acid (A). Positive ESI-MS profile of products of fermentation of *Moraxella* spp. MB1 without salicylic acid in biphasic medium (B). Positive ESI-MS profile of bioconversion products of salicylic acid (C).

**Figure 3:**
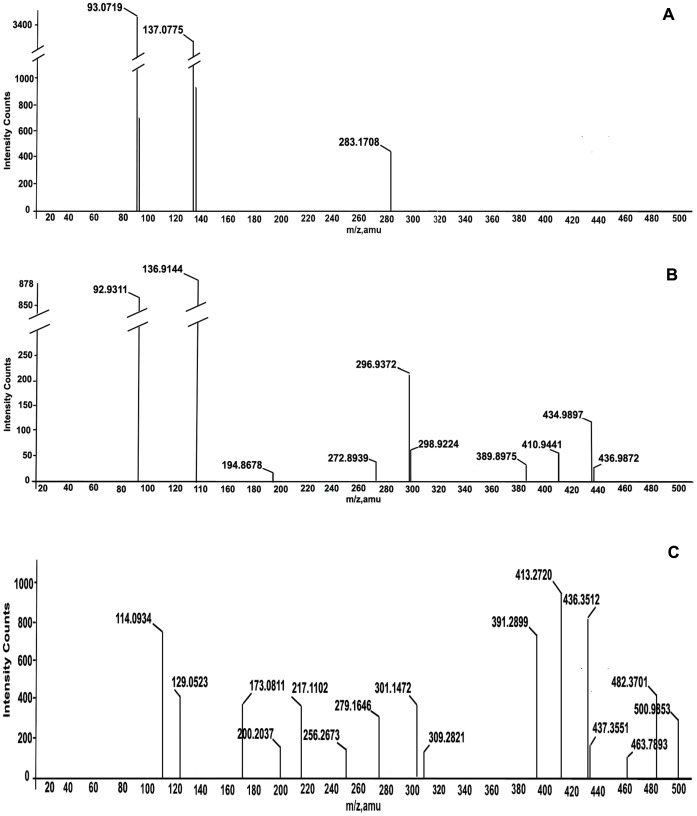
Negative ESI-MS profile of bioconversion products in methanol (A). Negative ESI-MS profile of bioconversion products in methanol+1% formic acid (B). Positive ESI-MS profile of ethyl acetate extract of symbiont *Bryopsis plumosa* (C).

ESI-MS is a very sensitive technique able to identify individual metabolites present in complex mixtures in picogram to fentogram range without prior purification. It provides information about the molecular mass and fragmentation pattern of the analyte. The mass spectra of phenolic acids were compared with those of the standards CA derivatives. Lacking commercial standards, p-coumaroyl, feruloyl, caffeoyl and trihydroxy-cinnamoyl conjugates were assigned by their parent ion and their spectroscopic fragmentation observed in both negative (−) and positive (+) ionization modes (Figure S1: A, C, Figure S2: B–F in File S1). Identification of compounds based on MS^2^ and MS^3^ analysis and comparison with literature, is discussed below and summarized in Table-1. As evident, positive ESI-MS (Figure 2C) is more sensitive technique than negative ESI-MS (Figure 3A, 3B) in detecting the compounds under the conditions used for analysis (Table-1). Among the identified compounds only three were detected in negative ESI-MS using methanol as solvent (Figure 3A), but, when the negative ESI-MS was recorded in methanol containing 0.1% formic acid it was much richer in deprotonated molecules, exhibiting 10 peaks (Figure 3B). In contrast, all the compounds were detected as protonated [M+H]^+^ molecular ions or as [M–H_2_O]^+^ ions in positive ESI-MS (Figure 2C).

**Table 1:** ESI-MS/MS characterization of metabolites in Ethyl Acetate extract of fermentation of *Moraxella* sp. MB1in biphasic medium containing SA: A-conjugates of SA with phenol and hydroxy cinnamic acids; B- Hydroxycinnamic acids derivatives produced by the bacterium; C-Standard Phenolic acids.

Compound identification	MW	MS (m/z)	MS^2^ fragmentation observed (m/z)
A: Conjugates			
SA (1)	138	137[M−H]^−^	93[Phenolate]^−^
Phenol (2)	94	93[M−H]	75[Phenyl]^−^
*p*-CoBA (3)	284	283[M−H]^−^	163 [Coumaratel]^−^
P-tri-OHCi (4)	272	273[M+H]^+^	179 [Trihydroxy cinnamoyl]^+^; 161 [Trihydroxy cinnamoyl – H_2_O]^+^; 143 [Trihydroxy cinnamoyl - 2H_2_O]^ +^; 197[Trihydroxy cinnamic acid +H]^ +^; 237 [M+H−2H_2_O]^ +^; 219[M+H−3H_2_O]^ +^
2-CBA (5)	300	301[M+H]^+^	283[M+H−H_2_O]^+^; 257[M+H−CO_2_]^+^; 239[M+H−(H_2_O+CO_2_)]^+^163[Caffeoyl] ^+^; 181[Caffeic acid+H]^+^
2-FBA (6)	314	315[M+H]^+^	177[Feruloyl]^+^; 195[Ferulic acid+H]^+^; 133[Ferulic acid+H−H_2_O−CO_2_]^+^; 297[M+H−H_2_O]^+^; 253[M+H−H_2_O−CO2]^+^
2-Tri-OH-CiBA (7)	316	317[M+H]^+^	179[Trihydroxy cinnamoyl]^+^; 197[Trihydroxy cinnamic acid +Hl]^+^; 299[M+H−H_2_O]^ +^; 271[M+H−HCOOH]^ +^; 219[M+H−3H_2_O−CO_2_]^ +^
p-CoBA MeEt (8)	298	299[M+H]^+^	281[M+H−H_2_O]^+^; 267[M+H−CH_3_OH]^+^; 255[M+H−CO_2_]^+^; 237[M+H−(H_2_O+CO_2_)]^+^; 219[M+H−(2H_2_O+CO_2_)]^+^161[p-methoxy cinnamoyl]^+^
B. Hydroxycinnamic acids derivatives			
p-Co-MeEt (9)	178	179[M+H]^+^	161[p-methoxycinnamoyl]^+^; 143[p-methoxycinnamoyl-H_2_O]^ +^; 135[M+H−CO_2_]^+^; 133[M+H−HCOOH]^+^;103[M+H−(CH_3_OH+CO_2_)]^+^
p-MeEt of Co-MeEs (10)	192	193[M+H]^+^	161[p-methoxycinnamoyl]^+^; 149[M+H−CO_2_]^+^;143[M+H−(CH_3_OH+H_2_O)]^+^; 133[M+H−(HCOOCH3)]^ +^;103[M+H−(OCH_3_+COOCH_3_)]^ +^
Methyl caffeate (11)	194	195[M+H]^+^	181[M+H−CH_3_]^ +^;177[M+H−H_2_O]^ +^;163[caffeoyl]^+^;151[M+H−CO_2_]^ +^; 149[M+H−HCOOH]^ +^;133[M+H−(H_2_O+CO_2_)]^+^
p-OH-styrene (12)	120	121[M+H]^+^	103[M+H−H_2_O]^+^; 93[Phenolate]^+^
C. Standard hydroxy CA			
Cinnamic acid	148	149[M+H]^+^	121[M+H−CO]^+^; 103[M+H−HCOOH]^+^; 77 [C_6_H_5_]^+^; 65[C_5_H_5_]
p-Coumaric acid	164	165[M+H]^+^	147[p-hydroxy cinnamoyl]^+^; 119[M+H−HCOOH]^+^; 77[C_6_H_5_]^+^
p-OMe-CA	178	179 [M+H]^+^	161[M+H−H_2_O]^+^;133[M+H−HCOOH]^+^;77[C_6_H_5_]^+^; 118[M+H−(HCOOH+CH_3_)]^+^;109[M+H−SC]^+^; 103[M+H−(CO_2_+OCH_3_]^+^
Caffeic acid	180	179[M−H]^−^	135[M−H− CO_2_]^−^; 89 [M−H−(CO_2_+HCOOH]^−^
Ferulic acid	194	193[M−H]^−^	178[M−H−CH_3_]^−^; 149[M−H−CO2]^_^; 134[M−H−(CO_2_+CH_3_)]^−^

SA, salicylic acid; P-phenol; p-CoBA, para-coumaroylbenzoic acid; P-tri-OHCi, phenyltrihydroxycinnamate; 2-CBA, 2-caffeoyl benzoic acid; 2-FBA, 2- feruloylbenzoic acid; 2-Tri-OH-CiBA, 2-trihydroycinnamoylbenzoic acid; *p*-CoBA MeEt, *para* -coumaroylbenzoic acid methyl ether; *p*-Co-MeEt, *para*-coumaric acid methyl ether; *p*-MeEt of Co-MeEs, *para*-methy ether of methylcoumarate; *p*-OMe-CA, *para*-methoxycinnamic acid.

### Identification of Metabolites Under Negative ESI-MS Conditions

As mentioned earlier, negative ESI-MS profile of bioconversion products (Figure 3A) displayed only three signals of which the signal at m/z 137 (Figure S1B in File S1) was due to the unreacted SA (1) and the remaining two anions were seen at m/z 93 and m/z 283. The ion at m/z 93, due to phenol (2) (Figure S1C in File S1), originated by decarboxylation of SA either by the bacterium [16] or by collision induced dissociation of unreacted SA. Negative ESI-MS/MS spectra of carboxylic acids (Table-1) are known to eliminate carbon dioxide (CO_2_) leading to the formation of [M−H]^−^ and [(M−H)−CO_2_] anions [17], [18]. Conjugate of phenol with trihydroxycinnamic acid (4) has also been identified as one of the bioconversion products of SA indicative of decarboxylation of SA to phenol.

The deprotonated [M−H]^−^ ion at m/z 283, gave single intense fragment at m/z 163 in its product ion mass spectrum (Figure S1A in File S1, Table-1) which suggested that it is an esterified form of coumaric acid [17] with SA. The diagnostic fragmentation pattern of coumaric acid conjugate involved cleavage of molecule into intact p-hydroxy cinnamyl and benzoic acid fragments. Therefore, the product is a conjugate of SA with coumaric acid, probably 2-(p- hydroxycinnamoyl) benzoic acid (3) and not 2-(hydroxycinnamoyl) salicylate because the expected fragment at m/z 239 [17], [18] due to elimination of CO_2_ was not observed. Probably intramolecular hydrogen bonding between the ortho positioned hydroxyl and carboxylic acid group affects the decarboxylation [19]. Further, in the positive ionization mode, all the biotransformed compounds identified are ortho-CA esters of benzoic acid. These observations led to propose structure (3) for the molecule with deprotonated [M−H]^−^ ion at m/z 283. Conjugate (3) appears as [M+H–H_2_O] at m/z 267 in positive full mass spectrum of the ethyl acetate extract and as methyl ether at m/z 299 (Figure 2C).

### Identification of Metabolites Under Positive ESI-MS Conditions

**Figure 4:**
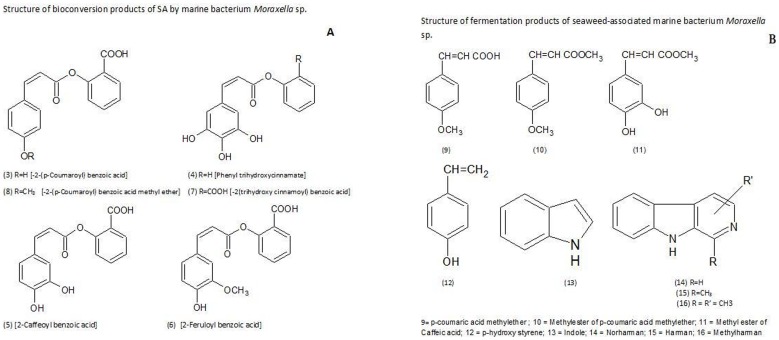
Structure of bioconversion products of salicylic acid by *Moraxella* spp. MB1 (A). Structure of fermentation products of seaweed associated *Moraxella* spp. MB1(B).

Positive ESI-MS profile (Figure 2C) besides protonated [M+H]^+^ ions, also displayed [M+H−H_2_O]^+^/[M+H−2H_2_O]^+^ peaks due to keto (site of protonation ) and hyroxyl groups in the benzene ring (loss of water). In addition, it also showed clear fragmentation patterns and the occurrence of common ion fragments (Figure 2C). Product ion spectra were recorded in order to identify classes of compounds and to obtain structural information of the compounds. However, from the intensity of the signal it cannot be determined if the greater intensity is attributable to higher concentration of the compound in the fermentation mixture or greater ionization efficiency of compounds. It should be noted that ions observed are likely to be mixture of isomers [18]. As the experiment involves bioconversion of SA, esterification probably occurs at the ortho position. ESI-MS profile of the bioconversion mixture (Figure 2C) in the positive ionisation mode yielded several peaks, each signal corresponded to protonated [M+H]^+^ molecular mass of individual molecular species. Each signal was subjected to product ion spectrum (MS^2^). The following conjugates of phenol and hydroxyl cinnamic acid derivatives with salicylic acid were identified (Figure 4A, 3–8) based on the fragmentation observed in their tandem mass spectra (MS/MS) (Figure S2: B–F in File S1, Table-1).

### Characterization of 2-caffeoyl Benzoic Acid (5)

Tandem Mass (MS^2^) spectrum of the [M+H]^+^ ion at m/z 301 (Figure S2B in File S1) produced the base fragment at m/z 283 by losing a water molecule [M+H−H_2_O]^+^ and a strong ion at m/z 257 [M+H−CO_2_]^+^ with the elimination of carbon dioxide. Simultaneous elimination of these two neutral molecules resulted in an intense signal at m/z 239. Caffeoyl ion was evident at m/z 163 [caffeoyl]^+^ whereas caffeic acid appeared as a small peak at m/z 181 [caffeic acid+H]^+^. Based on foregoing data the signal at [M+H]^+^301 was attributed to 2-caffeoyl benzoic acid (5).

### Characterization of 2-feruloyl Benzoic Acid (6)

Collision induced dissociation (CID) spectrum of the molecular species with [M+H]^+^ at m/z 315 (Figure S2C in File S1) exhibited base peak at m/z 177 due to loss of ferulic acid along with water which further loses CO_2_ to yield fragment at m/z 133. The presence of ferulic acid was evident by a small peak at m/z 195. The spectrum also displayed small ions at m/z 297 and 253 due to the loss of H_2_O and simultaneous elimination of H_2_O and CO_2_ respectively from the molecule. These data are in accordance with the structure of 2-feruloyl benzoic acid (6) for the molecule of [M+H]^+^at m/z 315.

### Characterization of Trihydroxycinnamates (4) and (7)

Targeted MS^2^ experiments at m/z at 273 (Figure S2D in File S1) and 317 (Figure S2E in File S1), the [M+H]^+^ ions of compounds 4 and 7 respectively produced a fragment at m/z 179 characteristic of trihydroxy cinnamoyl residue, and peak at m/z 197 due to the molecular ion of the corresponding acid. Elimination of a water and formic acid from compound 7 yielded the base fragment at m/z 299 [M+H−H_2_O]^+^ and a fragment at m/z 271 [M+H−HCOOH]^+^ respectively. An abundant fragment was also observed at m/z 219 due to simultaneous loss of 3H_2_O and a CO_2_ from the parent molecule. Similarly, in the product ion spectrum of compound 4 consecutive loss of one and two H_2_O molecules from the trihydroxy cinnamoyl moiety resulted in the base peak at m/z 161 and the fragment ion at m/z 143 respectively. Further, the conjugate 4 eliminates 2 and 3 water molecules to yield fragments at m/z 237 and 219 respectively. On the basis of foregoing data compound 4 was characterized as phenyl trihydroxycinnamate (4) a conjugate of trihydroxy cinnamic acid with phenol, and compound (7) as 2-(trihydroxycinnamoyl) benzoic acid (7) a conjugate of trihydroxy cinnamic acid with SA.

### Characterization of Methyl Ether of 2-(p-coumaroyl) Benzoic Acid (8)

The MS^2^ spectrum focused at m/z 299 yielded a fragment ion at m/z 161(Figure S2F in File S1) characteristic of p-methoxy cinnamoyl residue. This was further supported by the signals at m/z 281 and m/z 255 due to loss of water and CO_2_. Simultaneous elimination of water and carbon dioxide resulted in the product ion at m/z 237. Loss of two water molecules along with carbon dioxide yielded a fragment at m/z 219. Loss of methanol gave a signal at m/z 267. This compound is not an artefact of experimental procedure adopted as ethyl acetate was used for extraction.

**Figure 5:**
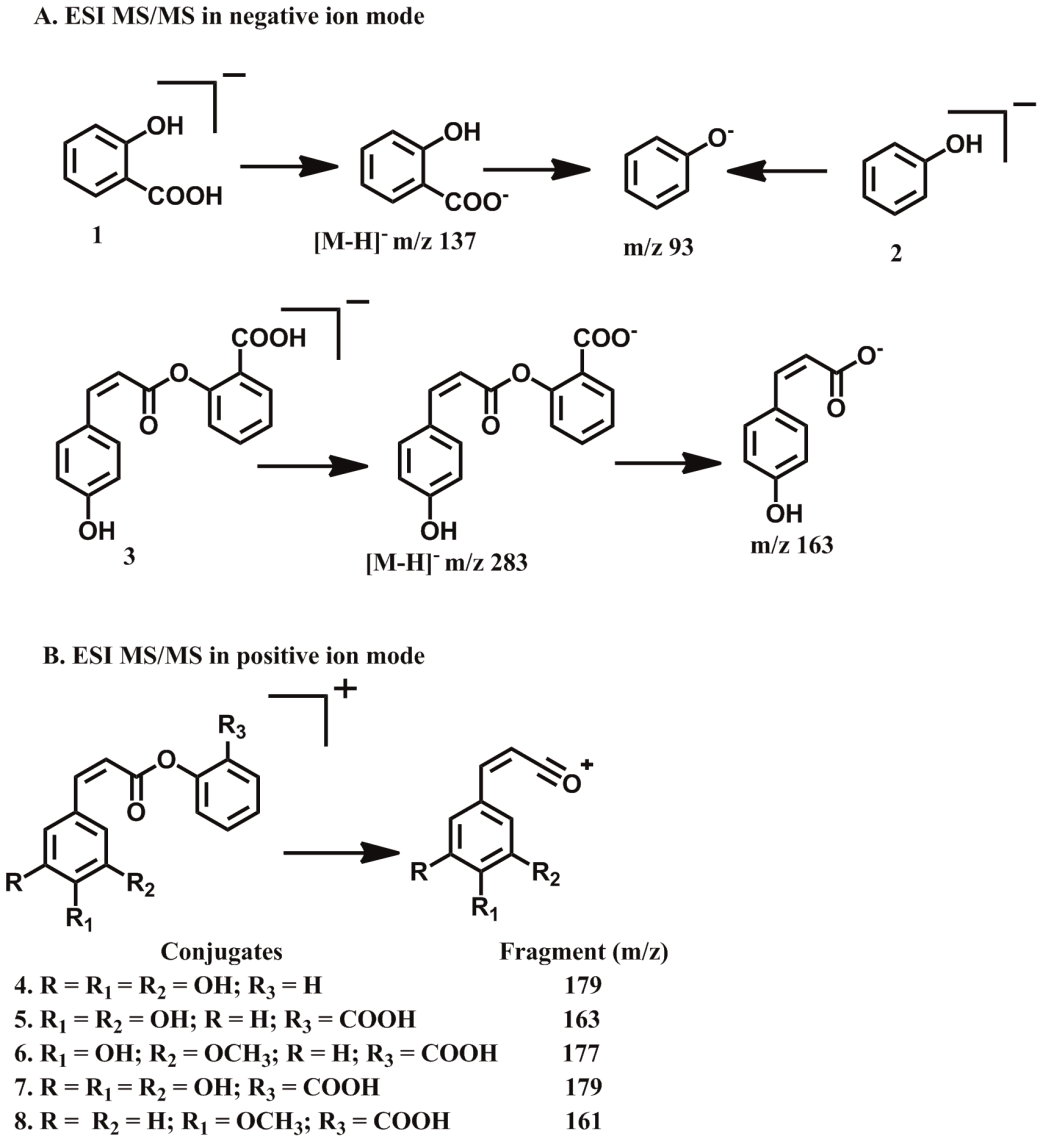
Proposed diagnostic mass spectral fragmentation of hydroxyl cinnamic acid esters. ESI-MS/MS in positive ionization mode (A); ESI-MS/MS in negative ionization mode (B).

In addition to the signals due to conjugates and their dehydration products (m/z 283, 281, 267, 255) several other signals probably due to hydroxyl cinnamic acids (9–12) and other metabolites (13–16) produced by the strain MB1, were also observed in full scan mass spectrum (Figure 2C) in positive ion mode of the products of biotransformation (Table-1). In analogy with literature reports [20], [21], the proposed fragmentation pathway involved in MS/MS spectra of these CA esters in positive as well as negative mode ionization is as depicted in (Figure 5A, 5B).

Decarboxylation of CA or its derivatives is expected to give styrene/styrene derivatives preferably when hydroxyl group is in 4-position (para). Positive ESI-MS profile of the crude ethyl acetate extract of bioconversion products indicated the presence of a compound with [M+H]^+^ at m/z 121 whose tandem mass spectrum (Figure S2A in File S1) is well in agreement with the structure of p-hydroxy-styrene (12, Figure 4B) derived from p-coumaric acid.

### Fermentation Products of *Moraxella* in Aqueous as well as Biphasic Media

The origin of hydroxyl cinnamic acid derivatives forming conjugates with SA was determined by cultivating *Moraxella* spp., strain MB1 under identical conditions but without the addition of SA in aqueous as well as biphasic medium. The metabolites were identified by ESI-MS (Figure 2A, 2B) and by TLC comparison with standards (Figure S3 in File S1). Analysis revealed that CA and its derivatives are in fact produced by the bacterium under aqueous as well as biphasic conditions as evident from the ESI-MS profile of the fermentation products of the bacterium (Figure 2A–C) which show signals for the presence of these compounds (MS^2^ spectra have not been included here but the fragmentation observed are presented in Table-1). ESI-MS profile and MS^2^ analysis (Figure S4 in File S1) was also indicative of the strain producing adenine (m/z 136), guanine (m/z 152), indole (m/z 118), β-carbolines (m/z 169, m/z 183 and m/z 197). The identification of these metabolites is based on the comparison of fragmentation observed in their tandem mass spectra with the literature reports [22], [23].

### Influence of Phenylalanine Addition to the Medium

Amino acid phenylalanine is a precursor of CA and it was found that there was enhancement in the production of phenolic acids on the addition of the amino acid to the cultivating media. This was evident from TLC analysis (Figure S3 in File S1).

### ESI-MS Analysis of Ethyl Acetate Extract of *Bryopsis plumosa*

To rule out the possibility of CA derivatives arising out of symbiotic association with the alga, *Bryopsis plumosa,* ESI-MS profile (Figure 3C) (Supriya Tilvi, Dissertation, 2005) of the ethyl acetate extract of the seaweed was examined. No signal for the protonated CA or hydroxyl-cinnamic acid derivatives was observed.

It is evident from the results of the present investigation that condensation reaction with elimination of water has taken place between salicylic acid and the fermentation products of marine bacterium, *Moraxella* MB1, hydroxyl cinnamic acid derivatives. There are several reports of similar condensation reactions leading to the production of bio-based polymers of commercial importance from microorganisms by fermentation. Typical examples are the family of polyesters, polyhydroxyalkanoates (PHA) that can be produced by various strains of bacteria, and the simplest one polyhydroxybutyrate was discovered as a constituent of bacterium *Bacillus megaterium* [24]. More than 150 PHA monomers have been identified as the constituents of PHAs [25]. Such diversity allows production of polymers with a wide range of properties tailored for specific applications. Pullulan is a linear water soluble polysaccharide mainly consisting of maltotriose units connected by α-1, 6 glycosidic units. It was first obtained from the fermentation broth of *Aureoasidium pullulans*. This polysaccharide is used as food additive, flocculant, blood plasma substitute, and as adhesives [26]. Alginate, a 1,4 linked linear polysaccharide of β-*d*-mannuronic and α–*l*-guluronic acid, well known as product of brown seaweeds is also biosynthesised by some bacteria, mostly derived from the genus *Pseudomonas* and belonging to the RNA superfamily I, and a soil bacterium *Azotobacter vinelandii* [27]. It is widely used as a gelling agent in pharmaceutical and food applications. Besides bio-based polymers of microbial origin, other well known natural polymers include proteins, nucleic acids, and polysaccharides (starch, cellulose, chitin and chitosan).

Generally, chemical condensation/polymerization between the two reactants involving removal of water molecule is a reversible reaction if not performed under anhydrous conditions. In nature, formation of bio-based polymers, in biological systems, are examples of *in vivo* enzymatic condensation reaction which are not affected by the presence of water. This may be due to the fact that water is indispensable for the change in chemical transformation that made life possible.

This study may be of direct application in preparative organic synthesis particularly condensation reactions involving removal of water. Enzymatic syntheses are preferred to chemical syntheses as the latter involves use of chemicals as well as formation of by products which are harmful as pollutants and needs rigorous treatment prior to disposal.

## Discussion

Bacteria belonging to genus *Moraxella* have been widely used for biotransformation. Gregg et al. [28] reports conversion of halo acetates to hydroxyl acetates catalyzed by fluoroacetate dehalogenase from *Moraxella* spp. B, while Spain et al. [29] described enzymatic substitution of nitro group by a hydroxyl group in p-nitrophenol degradation by a *Moraxella* strain. Yet another *Moraxella*, strain OA3, is known to degrade homogentisate, wherein, it is cleaved by a 1,2-dioxygenase yielding maleylacetoacetate [30]. There is a solitary report in the literature [15] on salicylate degradation via gentisate pathway by *Moraxella* strain VG45 isolated from oil field samples. *Moraxella* spp. MB1, strain of the present investigation, was reported earlier to decarboxylate nephrotoxin citrinin.

Results of the present investigation show that salicylic acid is not degraded by the seaweed associated marine bacterium *Moraxella* strain MB1 but undergoes esterification with hydroxycinnamic acid derivatives leading to the formation of conjugates of phenolic acids with salicylic acid (Figure 4A,Table-1). Hydroxycinnamic acids needed for condensation reaction are metabolites produced by *Moraxella* MB1 and do not arise from the symbiotic association of the strain with the seaweed, *Bryopsis plumosa* (Figure 3C). Though *Moraxella* spp. MB1 was reported earlier to decarboxylate nephrotoxin citrinin, in the present investigation it has been observed that in practically all the conjugates, except 4, the carboxyl group of salicylic acid moiety is intact probably because of intramolecular hydrogen bonding affects the decarboxylation. In decarboxylation of benzoic acid and CA and their hydroxyl derivatives by *Aerobacter aerogens* B-2614, it has been reported that enzyme activity requires a relatively unhindered 4-hydroxyl group on the aromatic ring and no decarboxylation is observed when the hydroxyl group is in ortho or para position when the reaction was carried out for 30 minutes [19]. The conjugates in the present study are not further metabolized as evident by the absence of signals in positive as well as negative ESI-MS profile of the bioconversion mixture (Figure 2C, Figures 3A and 3B) due to fission products of aromatic ring like 3-keto adipic acid (m/z 161), pyruvic acid (m/z 89), maleic/fumaric acids (m/z 117), succinic acid (m/z 119) and acetaldehyde (m/z 45). These products are expected when SA is degraded either via gentisic acid pathway or catechol pathway by scission of the benzene nucleus of catechol by 1,2/2,3 dioxygenase or of gentisate by gentisate 1,2 dioxygenase [15], [31], [32].

### Proposed Metabolic Pathway of Salicylic Acid Bioconversion Products

The degradation of aromatic compounds generally proceeds in two stages: ring substitution that results in the formation of dihydroxylated benzene derivatives (catechol, protocatechuate, gentisate, homogentisate and homoprotocatechuate) [33] followed by the degradation of the dihydroxylated benzene derivatives with ring fission and subsequent reactions linked to central metabolism in the cell. Ring fission is catalysed by ring cleavage dioxygenase that use molecular oxygen to open the aromatic ring between the two hydroxyl groups (ortho cleavage, catalysed by intra diol dioxygenases) [34] or proximal to one of the two hydroxyl groups (meta cleavage, catalysed by extradiol dioxygenases) [35].

**Figure 6:**
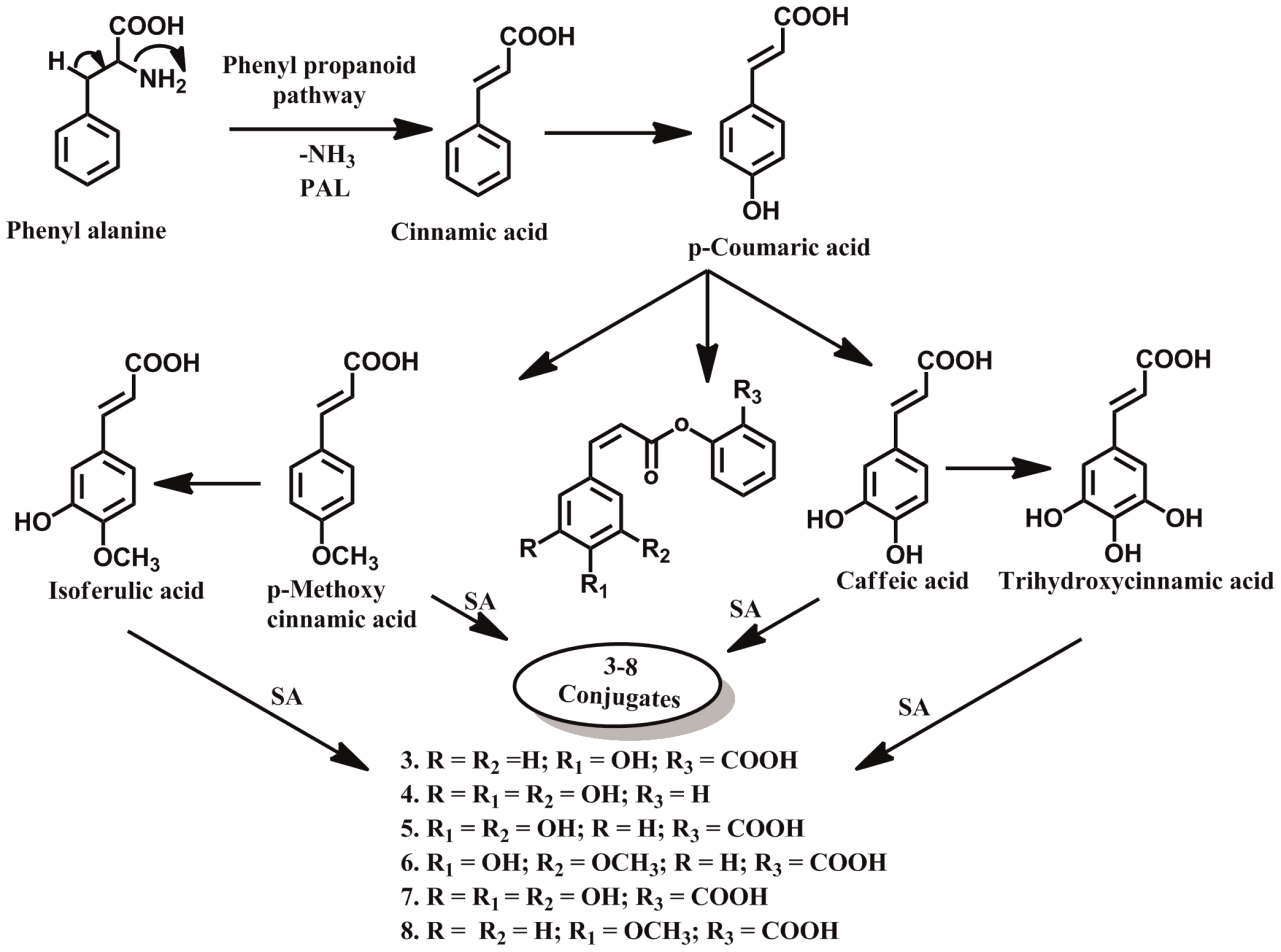
Scheme-1-Proposed metabolic pathway of formation of salicylic acid conjugates with hydroxycinnamic acid derivatives by *Moraxella* sp. MB1.

In analogy with literature [36] and the bioconversion metabolites identified, pathways involved in the metabolism of salicylic acid by marine bacterium *Moraxella* spp. MB1 have been envisaged as shown in Scheme-1(Figure 6). However, the various isoforms of the conjugates were not investigated in this communication because of the limitations of the technique.

Presumably, the bacterium synthesizes CA from phenylalanine by deamination, catalyzed by phenyl ammonia -lyase (PAL). PAL homologs are found in various plants, fungi, and yeasts but only in three prokaryotes *Streptomyces maritimus* [37], *Photorhadus luminescens* [38] and actinomycete *Saccharothrix espanaensis* [39]. Beginning with CA, a series of hydroxylation and methylation reactions catalyzed by enzymes hydoxylase and O-methyltransferase leads to the sequential synthesis of the common hydroxycinnamates [40]. Thus, hydroxylation of CA gives p-coumaric acid which on the introduction of second hydroxyl group gives caffeic acid and introduction of yet another hydroxyl group yields trihydroxycinnamic acid. Methylation of caffeic acid leads to the formation of ferulic acid while methylation of coumaric acid gives methyl ether of coumaric acid. These hydroxycinnamates are finally activated as hydroxycinnamate-CoA forms catalysed by hydroxycinnamate-CoA ligases. The activation of phenolic compounds as hydroxycinnamate-CoAs serve as building block molecules for further downstream modification which includes condensation and conjugation resulting in the biotransformation products of salicylic acid. This remains to be elucidated.

CA and its derivatives are secondary metabolites with antioxidants and antibacterial properties produced by plants in response to stressful conditions such as infections or wounding [41]. Fungi and yeast are also known to produce phenolic acids but as mentioned earlier PAL enzyme activity is rare in bacteria. CA possesses not only anti-tuberculosis properties [42] but is also known to inhibit the growth of several bacteria [43] in particular *Escherichia coli* O157:H7 [44]. It is also an active compound in medicinal plants with anti- *Helicobacter pylori* [45]. The bacterium of the present investigation besides CA derivatives, that forms conjugates with salicylic acid, also produces antimicrobial indole and β-carbolines, norharman, harman and its methyl derivative. All these compounds seem to play a protective role towards the host organisms and the environment.

Overall, the present study provides structural characterization by ESI-MS^n^ of hydroxycinnamates conjugates with SA as the metabolites of bioconversion of SA by marine bacterium *Moraxella* MB1. It also gives us information about the antimicrobial indole and β-carbolines as being the metabolites produced by the bacterium. These products are not only harmless to the environment but also play a protective role towards the host organisms. This study is of particular interest because SA makes its way into the sea, as pollutant, from different sources. Finally, we believe, this is a first report on the formation of conjugates of salicylic acid by a bacterium of genus *Moraxella.*

## Materials and Methods

No animal work has been conducted. All other work performed has been carried out according to the Institutional rules and regulations. The bacterial strain used for the study was isolated as an endobiont from the seaweed *Bryopsis plumosa* collected from Malvan (16°15′N, 17°338 35′E), Maharashtra coast, India, by the procedure described earlier [16]. Being an Oceanographic Institute, we are permitted to collect marine samples other than the endangered/protected species and samples from sensitive areas. The seaweed collected for the study was a very small quantity and do not belong to the category of protected species.

### Instrumentation

Nuclear Magnetic Resonance (NMR) spectra were recorded on a Bruker Avance AC 300 MHz instrument in deuterated chloroform (CDCl_3_) containing tetramethyl silane (TMS) as the internal standard. Mass spectra (ESI-MS) were acquired, in positive (+) as well as negative (−) ionization mode, using a QTOF-XL MS/MS, Applied Biosystem equipped with the MDS Sciex Analyst Software. The following setting was used for recording mass spectra so as to obtain optimum fragmentation: nebulizer gas (N_2_) 28 (arbitrary units); curtain gas (N2) 18 arbitrary units; ion spray voltage 5500 V for positive and −4500 V for negative mode, declustering potential (DP) 60.0; focusing potential (FP) 300 V; declustering potential (DP2) 15; and collision gas (CAD) 3 (arbitrary units). Full scan data acquisition was performed, scanning from m/z 0–400 in the profile mode and using a cycle time of 1 second. MS/MS were recorded at different collision energies in the range 10–40V. The identity of compounds was based on the fragmentation pattern observed and comparison with those of authentic standard cinnamic acid derivatives.

### Thin Layer Chromatographic Analysis

TLC analysis were performed on precoated kiselgel 60F_254_ (Merck) plates developed in petroleum ether: ethyl acetate: acetic acid (75∶25∶1) along with the authentic standards. Spots were visualized either by spraying with 5% methanolic sulfuric acid/alcoholic ferric chloride followed by heating at 100°C or by UV (ultraviolet) illumination under a dual wavelength (254 nm/330 nm) UV lamp.

### Bacterial Culture Isolation and Growth

The bacterial strain an endobiont from the seaweed *Bryopsis plumosa* collected from Malvan was maintained on nutrient agar slants which comprised of 5 g of peptic digest of animal tissue, 5 g of NaCl, 1.5 g of beef extract, 1.5 g of yeast extract and 20 g of agar dissolved in 1 litre of seawater-distilled water (1∶1, v/v), having pH 7.5 and stored at 4°C. The culture was screened for solvent tolerance by the procedure described by Ogino et al. [46]. The culture, with deposition no. NIOCC/OSTB-MB1 has been deposited at the Bioorganic Chemistry Laboratory of the National Institute of Oceanography, Goa, India.

### Identification of the Bacterium

Ethyl acetate solvent tolerant marine bacterium of the present investigation is a Gram negative, non-motile, cocco bacillus with cells as short rods, aerobic, oxidase and catalase positive and carbonic anhydrase negative. The culture did not produce acid from glucose and was found to be very sensitive to penicillin. On the basis of the above characteristics, the bacterium was identified as *Moraxella* spp. strain MB1. In addition, sequence analysis was carried out as described earlier [16]. Briefly, DNA was extracted from the stationary phase culture using a Bioron DNA isolation kit (Kit no. 501001). PCR amplification was performed in a total volume of 50 ml containing the appropriate reaction buffer and reagents and the universal primer 27 f (5′-GAGTTTGATCCTGGCTCA-3′) corresponding to *Escherichia coli* 16S rDNA numbering. The PCR conditions were as follows: initial denaturation (2 min at 95°C), followed by 30 cycles of denaturation (1 min at 95°C), primer annealing (1 min at 52°C), and primer extension (1.5 min at 72°C). The PCR amplification product was purified using a Qiagen kit (Kit no. 28104). The recovered fragment was sequenced using ABI 3700 Sequencer and partial 16SrRNA sequence was established as GGATGTTAGCGGCGGACGGGTGAGTACACGTGGGTAACCTGCCTGTAAGACTGGGATAACTCCG. The sequence obtained was subjected to BLAST search for closest match in the database. This bacterium showed 98% homology with the deposited sequence of uncultured marine bacterium clone ISA-3133 16S rRNA gene, partial sequence (535 bp), with GenBank accession no. AY936933.

### Growth of *Moraxella* spp. MB1 and Biotransformation of SA

The bacterium was grown in two flasks containing 100 ml of nutrient broth (Hi Media) at 28°C on a rotary shaker at 150 rpm whereas two uninoculated flasks with the same medium and under the same conditions served as control. Nutrient broth (Hi Media) comprised of 5 g of peptic digest of animal tissue, 5 g of NaCl, 1.5 g of beef extract, 1.5 g of yeast extract. After 8 hours, 200 mg of SA (Sigma) dissolved in 100 ml ethyl acetate was added aseptically to all the flasks and incubation continued for additional 40 hours. At the end of incubation period, the contents of the flasks were transferred to a separatory funnel, the organic layer separated from the biphasic cultivation system and the aqueous layer centrifuged (cell mass: 16 mg, dry weight) and the supernatant extracted thrice with ethyl acetate, the combined organic layer was washed with distilled water and dried over anhydrous sodium sulphate. Solvent from the extract was removed under vacuum on a rotavapor to yield a residue (43 mg/L, 21.5%) containing biotransformed products which were analysed spectrometrically using NMR (Figure 1B) and ESI-MS (Electrospray ionization mass spectra) data [Figure 2, 3; (Figure S1, S2 in File S1)].

### Fermentation of *Moraxella* spp. MB1 in Aqueous and Biphasic Media in the Absence of SA

The above experiment was repeated in aqueous and biphasic media without the addition of SA. After 48 hours of fermentation the contents were centrifuged at 10,000 rpm for 5 minutes (cell mass : 28 mg/L) and the supernatant worked out as described above to obtain ethyl acetate extract (80 mg/L, 40%) which was subjected to ESI-MS (Figure 2A, 2B) analysis in methanol and TLC (Figure S3 in File S1) using standards as reference compounds.

### Effect of Addition of Phenyl Alanine on the Fermentation of *Moraxella* spp

The marine bacterium, *Moraxella* MB1, was incubated at 28°C for 48 hours in nutrient broth (Hi Media) with the addition of phenylalanine (20 mg/L) a well known precursor of cinnamic acid (CA). At the end of the fermentation period the cells were harvested by centrifugation and the supernatant extracted with ethyl acetate and worked as mentioned earlier. The extract was spotted on TLC (Figure S3 in File S1) and the results (intensity of the spots) show that there is enhancement in the production of p-coumaric acid. Further evidenced by the fragmentation pattern observed using ESI-MS/MS conditions. Broth extract: 77 mg; cell mass: 26 mg).

## Supporting Information

File S1 **Combined Supporting Information File containing Figures S1–S4. Figure S1:** Negative ESI-MS/MS spectra of deprotonated [M−H]^−^ ions at m/z283 identified as 2-(hydroxycinnamoyl) benzoic acid (A); (m/z 137) unreacted salicylic acid; (m/z 93) phenol. **Figure S2:** Positive ESI-MS/MS spectra of protonated [M+H]^+^ ion at m/z 121 identified as p-hydroxystyrene (A); (m/z 301) 2-(caffeoyl) benzoic acid (B); (m/z 315) 2-(feruloyl) benzoic acid (C); (m/z 273) phenyl trihydroxycinnamate (D); (m/z 317) 2-(trihydroxycinnamoyl) benzoic acid (E); m/z 299 methyl ether of p-coumaroyl benzoic acid (F). **Figure S3:** TLC of metabolites produced under different conditions. *Moraxella* sp. MB1 grown in the presence of Phenylalanine [(A): Ethyl acetate extract E A (1), Methanol extract Cell mass (1)]; Control[Ethyl acetate extract E A (2), Methanol extract Cell mass (2)]. (B) Ethyl acetate extracts of broth of *Moraxella* sp. MB1 grown in the presence of solvent EA (3) and Control EA (4). **Figure S4:** Positive ESI-MS/MS of indole (m/z 118) and β-carbolines, norharman (m/z 169), harman (m/z 183) and methyl harman (m/z 197).
(ZIP)
