# Supplementary figures and images for: Fermentation Products of Solvent Tolerant Marine Bacterium Moraxella spp. MB1 and Its Biotechnological Applications in Salicylic Acid Bioconversion
Source: PLoS One. 2013 Dec 31;8(12):e83647. doi: 10.1371/journal.pone.0083647 (PMC3877071; doi:10.1371/journal.pone.0083647)

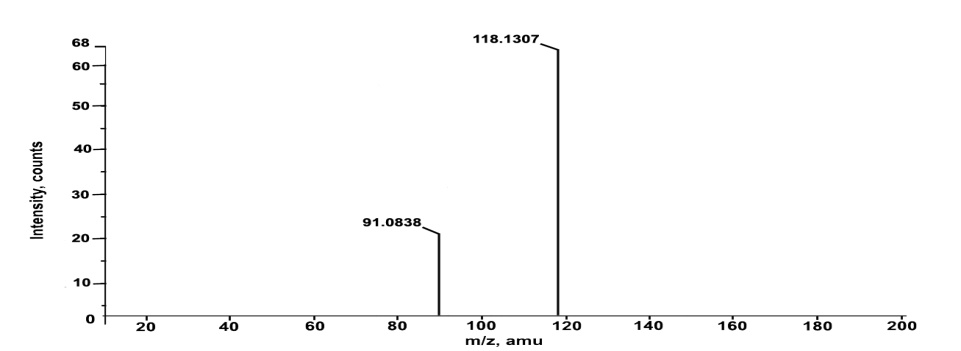


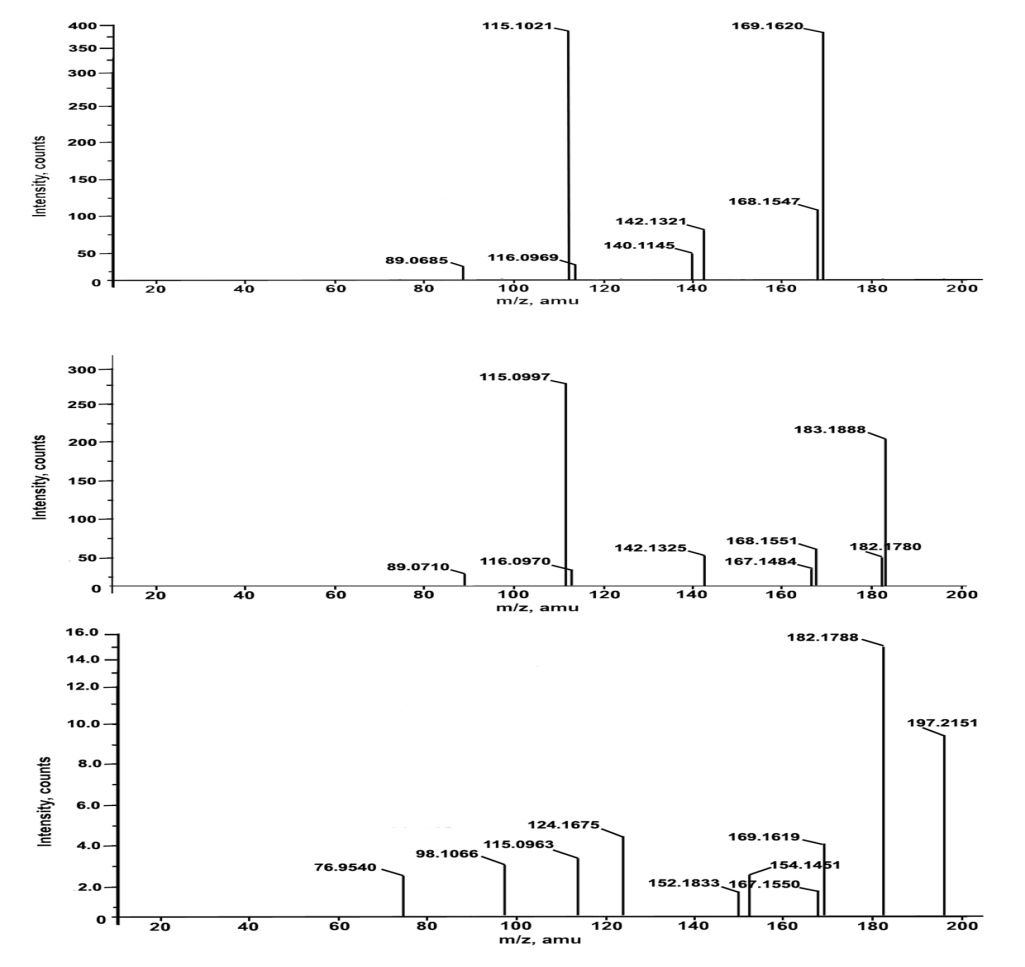

Supplement: File S1 — Combined Supporting Information File containing Figures S1–S4. Figure S1: Negative ESI-MS/MS spectra of deprotonated [M−H]− ions at m/z283 identified as 2-(hydroxycinnamoyl) benzoic acid (A); (m/z 137) unreacted salicylic acid; (m/z 93) phenol. Figure S2: Positive ESI-MS/MS spectra of protonated [M+H]+ ion at m/z 121 identified as p-hydroxystyrene (A); (m/z 301) 2-(caffeoyl) benzoic acid (B); (m/z 315) 2-(feruloyl) benzoic acid (C); (m/z 273) phenyl trihydroxycinnamate (D); (m/z 317) 2-(trihydroxycinnamoyl) benzoic acid (E); m/z 299 methyl ether of p-coumaroyl benzoic acid (F). Figure S3: TLC of metabolites produced under different conditions. Moraxella sp. MB1 grown in the presence of Phenylalanine [(A): Ethyl acetate extract E A (1), Methanol extract Cell mass (1)]; Control[Ethyl acetate extract E A (2), Methanol extract Cell mass (2)]. (B) Ethyl acetate extracts of broth of Moraxella sp. MB1 grown in the presence of solvent EA (3) and Control EA (4). Figure S4: Positive ESI-MS/MS of indole (m/z 118) and β-carbolines, norharman (m/z 169), harman (m/z 183) and methyl harman (m/z 197). (ZIP) [file pone.0083647.s001.zip › FigureS4.docx]

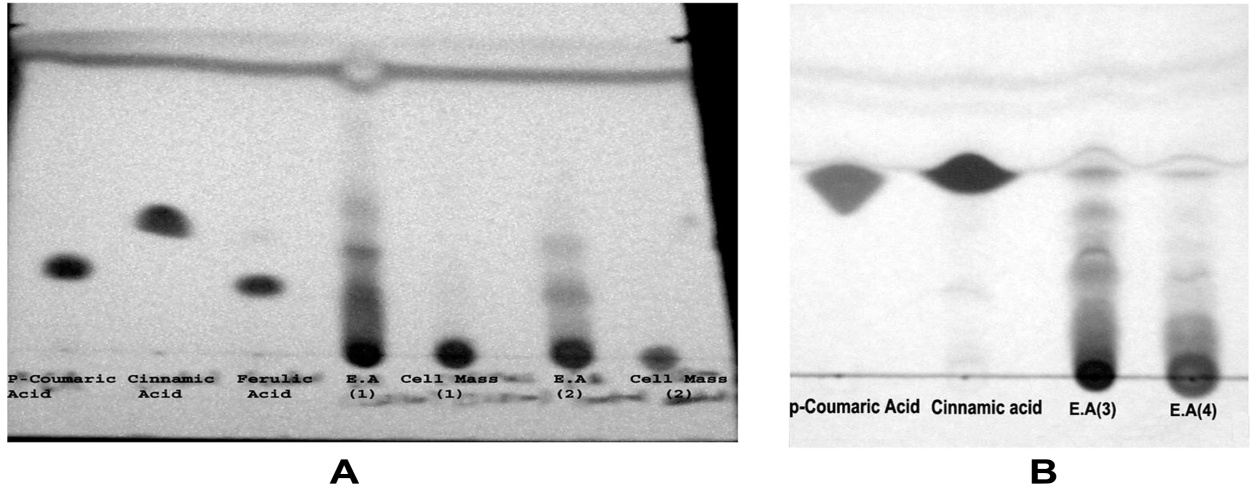

Supplement: File S1 — Combined Supporting Information File containing Figures S1–S4. Figure S1: Negative ESI-MS/MS spectra of deprotonated [M−H]− ions at m/z283 identified as 2-(hydroxycinnamoyl) benzoic acid (A); (m/z 137) unreacted salicylic acid; (m/z 93) phenol. Figure S2: Positive ESI-MS/MS spectra of protonated [M+H]+ ion at m/z 121 identified as p-hydroxystyrene (A); (m/z 301) 2-(caffeoyl) benzoic acid (B); (m/z 315) 2-(feruloyl) benzoic acid (C); (m/z 273) phenyl trihydroxycinnamate (D); (m/z 317) 2-(trihydroxycinnamoyl) benzoic acid (E); m/z 299 methyl ether of p-coumaroyl benzoic acid (F). Figure S3: TLC of metabolites produced under different conditions. Moraxella sp. MB1 grown in the presence of Phenylalanine [(A): Ethyl acetate extract E A (1), Methanol extract Cell mass (1)]; Control[Ethyl acetate extract E A (2), Methanol extract Cell mass (2)]. (B) Ethyl acetate extracts of broth of Moraxella sp. MB1 grown in the presence of solvent EA (3) and Control EA (4). Figure S4: Positive ESI-MS/MS of indole (m/z 118) and β-carbolines, norharman (m/z 169), harman (m/z 183) and methyl harman (m/z 197). (ZIP) [file pone.0083647.s001.zip › Figure S3.docx]

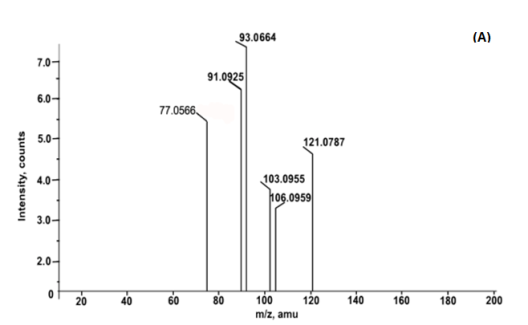

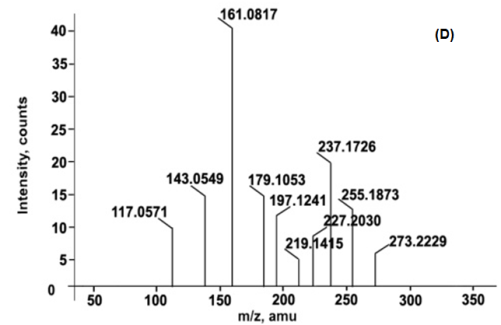


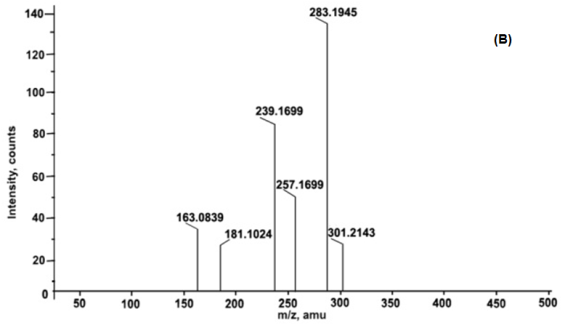

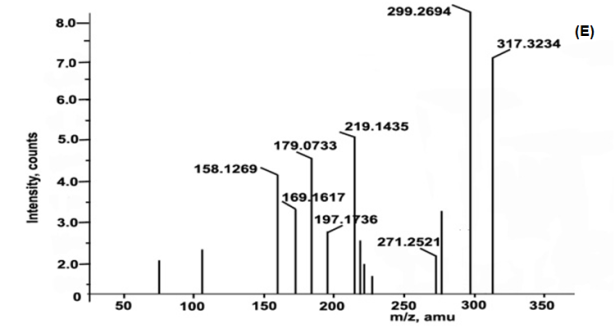


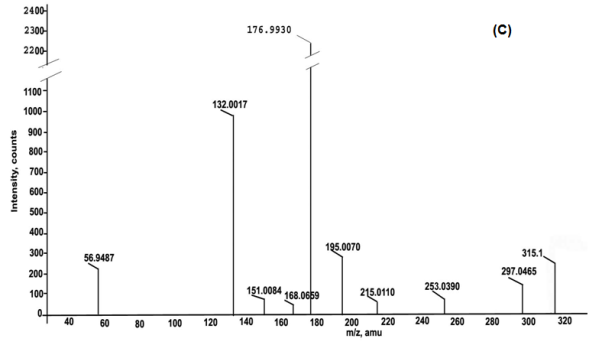

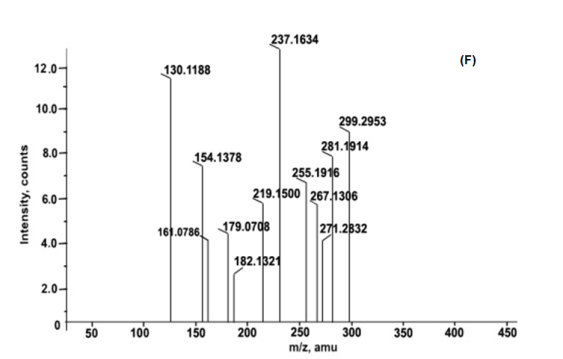

Supplement: File S1 — Combined Supporting Information File containing Figures S1–S4. Figure S1: Negative ESI-MS/MS spectra of deprotonated [M−H]− ions at m/z283 identified as 2-(hydroxycinnamoyl) benzoic acid (A); (m/z 137) unreacted salicylic acid; (m/z 93) phenol. Figure S2: Positive ESI-MS/MS spectra of protonated [M+H]+ ion at m/z 121 identified as p-hydroxystyrene (A); (m/z 301) 2-(caffeoyl) benzoic acid (B); (m/z 315) 2-(feruloyl) benzoic acid (C); (m/z 273) phenyl trihydroxycinnamate (D); (m/z 317) 2-(trihydroxycinnamoyl) benzoic acid (E); m/z 299 methyl ether of p-coumaroyl benzoic acid (F). Figure S3: TLC of metabolites produced under different conditions. Moraxella sp. MB1 grown in the presence of Phenylalanine [(A): Ethyl acetate extract E A (1), Methanol extract Cell mass (1)]; Control[Ethyl acetate extract E A (2), Methanol extract Cell mass (2)]. (B) Ethyl acetate extracts of broth of Moraxella sp. MB1 grown in the presence of solvent EA (3) and Control EA (4). Figure S4: Positive ESI-MS/MS of indole (m/z 118) and β-carbolines, norharman (m/z 169), harman (m/z 183) and methyl harman (m/z 197). (ZIP) [file pone.0083647.s001.zip › Figure S2.docx]

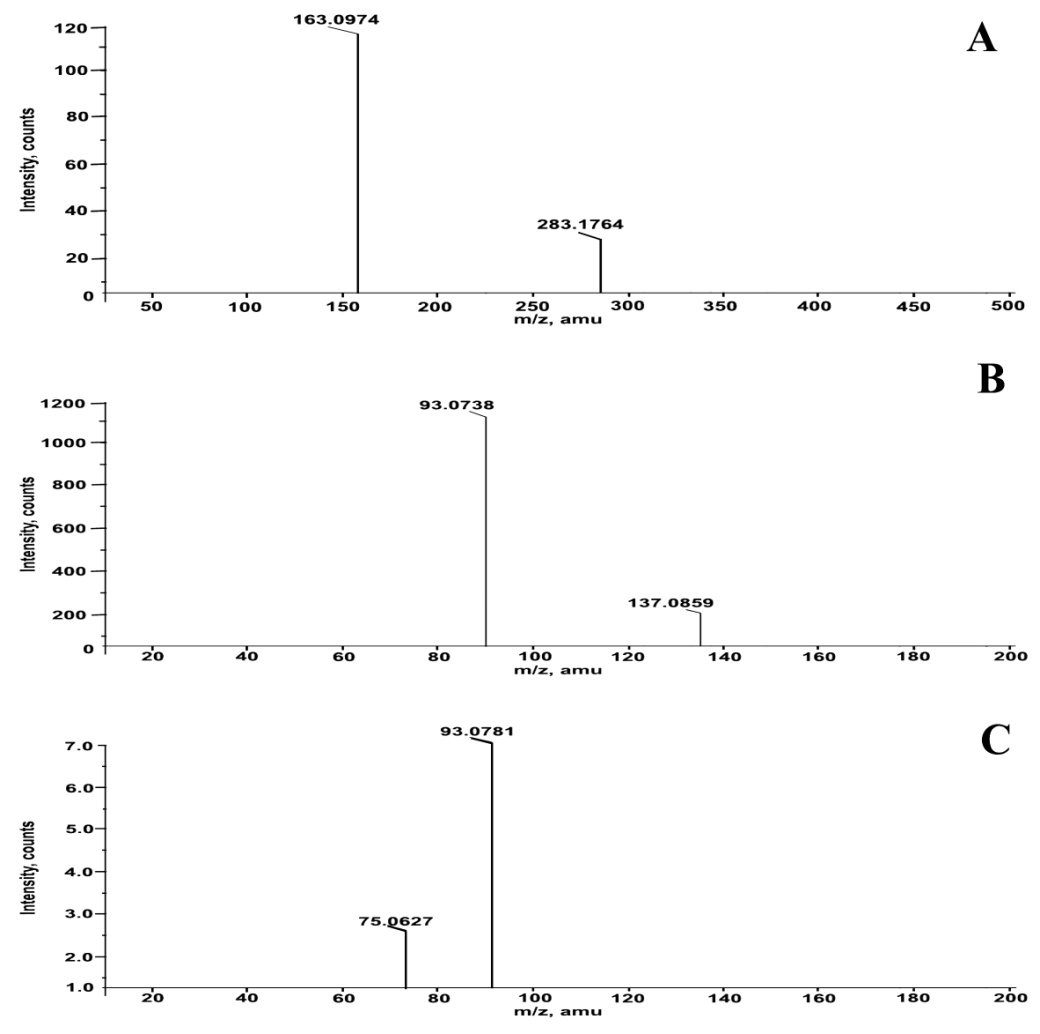

Supplement: File S1 — Combined Supporting Information File containing Figures S1–S4. Figure S1: Negative ESI-MS/MS spectra of deprotonated [M−H]− ions at m/z283 identified as 2-(hydroxycinnamoyl) benzoic acid (A); (m/z 137) unreacted salicylic acid; (m/z 93) phenol. Figure S2: Positive ESI-MS/MS spectra of protonated [M+H]+ ion at m/z 121 identified as p-hydroxystyrene (A); (m/z 301) 2-(caffeoyl) benzoic acid (B); (m/z 315) 2-(feruloyl) benzoic acid (C); (m/z 273) phenyl trihydroxycinnamate (D); (m/z 317) 2-(trihydroxycinnamoyl) benzoic acid (E); m/z 299 methyl ether of p-coumaroyl benzoic acid (F). Figure S3: TLC of metabolites produced under different conditions. Moraxella sp. MB1 grown in the presence of Phenylalanine [(A): Ethyl acetate extract E A (1), Methanol extract Cell mass (1)]; Control[Ethyl acetate extract E A (2), Methanol extract Cell mass (2)]. (B) Ethyl acetate extracts of broth of Moraxella sp. MB1 grown in the presence of solvent EA (3) and Control EA (4). Figure S4: Positive ESI-MS/MS of indole (m/z 118) and β-carbolines, norharman (m/z 169), harman (m/z 183) and methyl harman (m/z 197). (ZIP) [file pone.0083647.s001.zip › Figure S1.docx]
